# Left atrial volumes and associated stroke subtypes

**DOI:** 10.1186/1471-2377-13-149

**Published:** 2013-10-18

**Authors:** Quratulain Shaikh, Bilal Ahmed, Maryam Ahmed, Jamal Hussain Mahar, Masood Ahmad, Ayesha Ahmed, Farzin Majeed, Fariha Sadiq Ali, Maria Khan, Ayeesha Kamran Kamal

**Affiliations:** 1 Neurovascular Clinical Research Fellow, The International Cerebrovascular Translational Clinical Research Training Program, Stroke Services, Aga Khan University, Karachi, Pakistan; 2 Department of Medicine, Aga Khan University, M Sc. Epidemiology and Biostatistics, Karachi, Pakistan; 3 Stroke Service, Aga Khan University, Karachi, Pakistan; 4 Stroke Research Electives, Medical College, Aga Khan University, Karachi, Pakistan; 5 Section of Cardiology, Department of Medicine, Aga Khan University, Karachi, Pakistan; 6 The International Cerebrovascular Translational Clinical Research Training Program, Stroke Services, Aga Khan University, Karachi, Pakistan; 7 Stroke Services, Section of Neurology, Department of Medicine, Stroke Fellowship Program, International Cerebrovascular Translational Clinical Research Training Program, Aga Khan University Hospital, Stadium Road, 74800, Karachi, Pakistan

## Abstract

**Background:**

Cardio embolism and cerebrovascular atherosclerosis are two major mechanisms of stroke. Studies investigating associations between advanced echocardiographic parameters and stroke mechanisms are limited.

**Methods:**

This study is a standardized review of 633 patients admitted to the stroke service of a tertiary care hospital following a standardized stroke investigation and management pathway. Stroke subtypes were characterized using the Causative Classification System, using the hospitals online radiologic archival system with CCS certified stroke investigators. Patients with two mechanisms were excluded.

**Results:**

Patients with cardioembolic stroke had a higher proportion of atrial fibrillation (p < 0.001), acute myocardial infarction (p < 0.001) and ischemic heart disease (p < 0.001). On electrocardiogram (ECG) and transthoracic Echo (TTE), patients with cardioembolic stroke had a greater atrial fibrillation (p < .00), left ventricular thrombus (p < .00), left ventricular ejection fraction <30% (p < .00) and global hypokinesia (p < .00) Patients with cardioembolic stroke had higher mean left atrial volume indices (LAVi) (p < 0.001), mean left ventricular mass indices (LVMi) (p < 0.05) and mean left atrial diameters (LAD) (p < 0.05). At LAVi of 29–33 ml/m^2^, the risk of atherothrombotic stroke increased. The risk of cardioembolic stroke increased with LAVi of 34 ml/m^2^ and above.

**Conclusion:**

Left atrial volume indices may be linked to specific stroke phenotype. At mild increases in left atrial dimensions, the risks of atherosclerotic stroke are high, and probably reflect hypertension as the unifying mechanism. Further increases in left atrial dimensions shifts the risk towards cardioembolic stroke.

## Background

Strokes constitute a global health epidemic and are the leading cause of sustained disability [1]. Ischemic stroke is a heterogeneous disease, the two main subtypes being cardioembolic stroke and atherosclerotic stroke [2]. It is important to be able to identify these subtypes since therapeutic decisions for future prevention may differ [3]. The underlying pathological processes are different in the two subtypes; while advanced atherosclerotic stenosis is deemed to be the major mechanism in atherothrombotic stroke, it is the underlying cardiac abnormalities with relatively preserved architecture of blood vessels that eventually manifests as cardioembolic strokes.

Atherosclerosis leading to hypertension is mirrored on an echocardiogram as left ventricular hypertrophy [4]. Historically, echocardiographic predictors of vascular outcome have focused on left ventricular morphology. Left ventricular mass was found to predict stroke and coronary artery disease [5]. In the Framingham Study, for each 50-g increase in LVM, there was 1.5-fold increase in relative risk for subsequent cardiovascular events [6]. Recent studies have reported left atrial diameter and left atrial volume index to be associated with chronic hypertension and stroke [7]. Although these values have helped in predicting ischemic stroke their association with specific stroke subtypes has not been established.

We hypothesized that patients who present with cardioembolic stroke have greater Left Atrial Diameters (LAD), increased Left Atrial Volume Indices (LAVi) and Left Ventricular Mass (LVM) when compared to those with atherosclerotic stroke. We investigated the relationship if any, between atrial predictors of stroke subtype on routine post stroke transthoracic echocardiograms.

## Methods

The Aga Khan University Hospital is a JCIA (Joint Commission International Accreditation) accredited tertiary care hospital in Karachi, Pakistan. Stroke care is delivered through a 24-hour on-call neurology team with a stroke service on call for hyperacute stroke with a 5 bed specialized stroke unit and a full range of ancillary services. As a standard of care, all admitted stroke patients are evaluated by a consultant neurologist and undergo stroke workup that includes neuroimaging via MRI and MRA, electrocardiogram (ECG), transthoracic echocardiography (TTE), Carotid Doppler Sonography and Biochemical workup such as serum glucose, lipid profile, blood urea nitrogen and serum creatinine. These are standardized investigations that are conducted on every admission and ensure homogeneity of evaluation. All these scans and echocardiograms are accessible via the hospital online database that details all investigations for these patients. In addition, all admissions are encoded for stroke diagnosis through a hospital wide ICD-9 coding system allowing identification of all admissions.

This set up enabled a comprehensive review of all adult stroke patients admitted to this service from July 2005 to December 2010. There were 633 patients admitted, identified as suffering from ischemic stroke through the ICD-9 code with the assistance of medical records. Patients with evidence of hemorrhagic stroke, subarachnoid hemorrhage, and those with hematologic, neoplastic, infectious, iatrogenic or inflammatory conditions associated with stroke were excluded because the pathophysiology and natural course of these causes differ from atherosclerotic ischemic stroke. Patients with stroke due to any other subtype e.g. Cryptogenic stroke, stroke due to known hypercoagulable state, dissection were excluded from this review. Additionally, patients with more than one competing diagnosis were excluded.

Data was reviewed for demographic characteristics and electrocardiographic and echocardiographic data was also recorded on a predesigned structured questionnaire. This was developed in conjunction with cardiological input.

Demographic variables included age and gender, medical variables including height, weight, history or evidence of diabetes mellitus, hypertension, dyslipidemia, history of ischemic heart disease, prior stroke, and history of atrial fibrillation, congestive cardiac failure and tobacco use were recorded. ECGs were analyzed for evidence of atrial fibrillation. Echocardiographic evidence was documented for the presence of left atrial thrombus, left ventricular thrombus, presence of any vegetation, global segmental hypokinesia, left ventricular ejection fraction less than 30%, measurements were recorded for left atrial diameter (LAD) which was measured in antero posterior (AP) linear dimension obtained from the parasternal long axis view, at Left Ventricular end systole (when left atrium is at the maximum volume), left atrial volume index (LAVi), left ventricular diameter in diastole (LVD), posterior left ventricular wall thickness (PWT), and interventricular septal thickness(IVST). Left Ventricular mass (LVM) was calculated using the guidelines from American Society of Echocardiography (ASE) and indexed for body surface area (LVMi) as recommended for reference in population. Reference values for LAD, LAVi and LVMi in males and females were derived from Guidelines from the American Society of Echocardiography [8].

Furthermore for left atrial measures, since single axis measurement is unreliable when atrial dilation is non-uniform, in our laboratory we actually measure both. i.e. the LA diameter in AP length and also the atrial volume which is measured by using area-length (L) method using Apical 4 chamber view (A1) and apical 2 chamber view (A2) at ventricular end systole. The information is put in the formula 0.85 (A1 × A2) divided by L for the atrial volume and indexed to Body Surface Area (BSA) giving us the left atrial volume index (LAVi).

Strokes were classified using the Causative Classification of Stroke by neurologists who were trained in stroke diagnosis and ran a stroke clinic [9]. In addition, magnetic resonance imaging and carotid doppler scans read by trained neuroradiologists were utilized for this classification. The stroke classifying personnel were trained and certified in this technique before undertaking this review. This classification team did not have access to the exact advanced echocardiographic measures that are reported in this study during the time of stroke classification. The CCS system is an online system where data is entered into a computerized system and it assists in the correct mechanistic diagnosis, with a level of confidence assigned to the mechanism as probable, possible or evident, we included patients in the cardioembolic and atherosclerotic stroke category when any of these levels were assigned, and there were no competing mechanisms.

After entering the data for stroke classification, demographic variables and echocardiographic data, simple frequencies were run and comparisons were performed. Data was entered centrally with the echocardiography team and classification team both unaware of any emerging trends till the data entry was complete.

For the purpose of this comparative analysis those with evident, possible and probable Cardioembolic Strokes were compared with patients with stroke due to atherosclerotic mechanisms (Large artery, ICAD -**I**ntra**C**ranial **A**therosclerotic **D**isease, ECAD-**E**xtra**C**ranial **A**therosclerotic **D**isease and Small vessel strokes).

The study was approved by the Ethical Review Committee of the Aga Khan University through approval number 1660-Neu-ERC-2010 under exemption category as it is a detailed review.

### Statistical analysis

Data were analyzed through SPSS 19, and reported as mean ± SD for continuous variables and proportions and percentages for categorical data. Comparisons between groups were done by the unpaired Student’s t-test and Chi- square test respectively. P value less than 0.05 was considered significant. Normality of the data was assessed by Kolmogorov-Smirnov statistic.

## Results

There were 633 patients included in this study. All patients had undergone ECG, MRI, DWI, MRA, TTE and carotid doppler. Of these 60% were in the atherosclerotic ischemic stroke group and 40% were cardioembolic ischemic stroke group. 64% of the patients were male. The median age was 61 years. According to the Asia Pacific criteria for obesity 56% of the patients were obese [10]. 79% of the patients were hypertensive and 22% had a prior stroke. 17% were using tobacco (Table 1).

**Table 1:** Baseline demographic characteristics of patients by stroke subtype

	**All stroke patients**	**Cardioembolic stroke group**	**Atherosclerotic stroke group**	**P value**
	**N 633**	**N 256 (40)**	**N 377 (59.9)**	
**Gender men/women**	406 (64)	163 (40)	243 (59.9)	0.45
BMI obese^*^	356 (56.2)	142 (40)	214 (60)	0.76
**Ischemic heart disease**	142 (22.4)	77 (54.2)	65 (45.8)	0.00
**Previous MI**	47 (7.4)	25 (53.2)	22 (46.8)	0.04
**Acute MI**	18 (2.8)	13 (72.2)	5 (27.8)	< .00
**Type II DM**	325 (51.3)	132 (40.6)	193 (59.4)	0.49
**Hypertension**	500 (79)	201 (40.2)	299 (59.8)	0.44
**Cardiac valve prosthetic**	9 (1.4)	7 (77.8)	2 (22.2)	0.02
**Atrial fibrillation**	51 (8.1)	40 (78.4)	11 (21.6)	0.00
**Valvular heart disease**	5 (0.8)	4 (80)	1 (20)	0.09
**Dyslipidemia**	336 (53)	128 (38)	208 (61.9)	0.11
**Tobacco use**	109 (16.9)	45 (42)	62 (57.9)	0.39
**Prior stroke**	145 (22.9)	57 (39.3)	88 (60.7)	0.41
**Heart failure**	57 (1.1)	5 (71.4)	2 (28.6)	0.09

*Asia Pacific Criteria for obesity as applicable to South Asians is used to define this population.

Legend: Patients with cardioembolism have more ischemic heart disease, heart failure, valvular heart disease and replacements, atrial fibrillation as compared to patients with atherosclerotic stroke.

Patients with cardioembolic stroke had a significantly higher prevalence of atrial fibrillation (p < .00), acute myocardial infarction (p < .00) and history of ischemic heart disease (p < .00). They were more prone to develop acute myocardial infarction (MI) during hospital stay and had significantly higher prevalence of previous myocardial infarction. Moreover the history of cardiac valve replacement was significantly higher in the cardioembolic stroke sub population. Rest of the demographic variables was comparable in the two groups.

ECG and TTE data revealed that cardioembolic stroke group had higher prevalence of atrial fibrillation (p < .00), left ventricular thrombus (p < .00), left ventricular ejection fraction < 30% (p < .00) and global ventricular hypokinesia (p < .00) (Table 2).

**Table 2:** Yield of conventional electrocardiographic and transthoracic echocardiographic evaluation of patients by stroke subtype

**Finding on ECG**^**#**^/Echocardiogram*	**All stroke patients**	**Cardioembolic stroke group**	**Atherosclerotic stroke group**	**P value**
	**n (%)**	**n (%)**	**n (%)**	
**Atrial fibrillation**^**#**^	56 (8.8)	39 (69.6)	17 (30.4)	< .00
**Atrial flutter**^**#**^	3 (0.5)	3 (1.2)	0 (0)	0.06
**Evidence of prior MI**^**#**^	37 (95.8)	19 (951.4)	18 (48.6)	0.11
**First degree AV block**^**#**^	46 (7.3)	22 (47.8)	24 (52.2)	0.18
Left ventricular thrombus*	15 (2.4)	12 (80)	3 (20)	< .00
Left atrial thrombus*	1 (0.2)	0 (0)	1 (0.3)	0.59
Vegetation*	1 (0.2)	0 (0)	1 (0.3)	0.59
LVEF < 30%*	68 (10.7)	56 (82.4)	12 (17.6)	< .00
Global segmental hypokinesia*	108 (17.1)	74 (68.5)	34 (31.5)	< .00

Legend: Atrial flutter and fibrillation on 12- Lead EKG were clearly helpful, although low yield overall at 8.8% of the entire study group (56/633), Obvious echocardiographic predictors mostly ventricular based like thrombi, vegetations, EF reduction, and hypokinesia were actually also low yield.

# Findings on ECG.

* Findings on Echo cardiogram.

Patients with cardioembolic stroke had higher mean left atrial volume indices (LAVi) (p < .00), mean left atrial diameters (LAD) (p < .05) and left ventricular mass indices (LVMi) (< .05) than the atherothrombotic stroke patients (Table 3).

**Table 3:** Trends of left atrial enlargement and stroke subtypes

		**LA diameter males**	**LA diameter females**	**LAVi males**	**LAVi females**
**Cardioembolic stroke**	**None**	138 (85)	65 (70)	54 (52)	19 (31)
	**Mild**	9 (6)	8 (9)	18 (17.5)	16 (26)
	**Moderate**	8 (5)	11 (12)	14 (14)	11 (18)
	**Severe**	7 (4)	9 (10)	17 (16.5)	16 (25.5)
**Atherothrombotic stroke**	**None**	206 (84)	109 (81)	87 (52)	39 (44)
	**Mild**	28 (11.5)	14 (10)	42 (25)	25 (28)
	**Moderate**	8 (3)	3 (2)	25 (15)	14 (16)
	**Severe**	2 (0.8)	8 (6)	13 (8)	10 (11)
**P value**		< .020	< .016	< .105	< .098

Legends: **LA Diameter**: Left atrial Diameter.

**LAVi**: Left atrial volume index.

At LAVi of 29–33 ml/m^2^, the risk of atherothrombotic stroke increased. The risk of cardioembolic stroke increased with LAVi of 34 ml/m^2^ and above. Hence the observation was that the risk of cardioembolic stroke increased with increasing left atrial volume indices in the moderate to severely enlarged category. At mild increase in left atrial diameter and left atrial volume index the risk of atherosclerotic stroke was increased (Table 4).

**Table 4:** Distribution of left atrial enlargement and left ventricular hypertrophy by stroke subtype

**Mean Values**	**Cardioembolic stroke**	**Atherothrombotic stroke**	**P value**
LAVi^*^	33.4	30	< .00
**ml/m**^**2**^			
**LAD**^**#**^	36	35	< .05
**mm**			
**LVMi**^**ß**^	80.9	76	< .05
**gm/m**^**2**^			

^*^LAVi: Left atrial volume index, left atrial diameter and left atrial volume indexed to body surface area is a better measure when left atrial dilation is non- uniform.

^**#**^**LAD:** Left atrial dilation is a single-axis measurement based on the antero-posterior length of the left atrium, may be unreliable when dilation is non-uniform.

^**ß**^**LVMi:** Left ventricular mass index, Left ventricular mass indexed to body surface area is a measure of the left ventricular hypertrophy.

## Discussion

Although transthoracic echo is a routine investigation in stroke patients, its value for predicting stroke sub typing has been relatively limited as all the data contained in these studies is often inadequately utilized by neurologists. In another study from this region, the yield for predicting stroke subtype was reported to be approximately 15% for cardioembolic sources of ischemic stroke [11]. We believe that this is the first detailed report on the putative mechanisms and advanced echocardiographic associations of stroke subtypes, which report that atrial dilation initially associates with atherosclerotic strokes and later with further increases, associates with cardioembolism. Conventionally, echocardiographic measures have focused on ventricular predictors of cardioembolism and have failed to appreciate atrial predictors. Data deeming atrial fibrillation and atrial flutter as risk factors for cardioembolic stroke has been validated by many investigators Other well appreciated and mostly ventricular risk factors for cardioembolic stroke that have been identified include acute myocardial infarction, history of prior MI, history of cardiac valve replacement and history of ischemic heart disease that affect valvular parameters or ventricular hypokinesia.

There is adequate data reporting left atrial volume index (LAVi) to be a better measure of left atrial dilatation as compared to left atrial diameter, providing a more accurate assessment of left atrial size than conventional M-mode LAD [7-9]. LAD is a single-axis measurement based on the antero-posterior length of the left atrium. When left atrial enlargement is not uniform, LAD will therefore, not reflect left atrial size accurately. Recent guidelines from the American Society of Echocardiography (ASE) have recommended LAVi measurement be used for left atrial size quantification in clinical practice [8]. Left atrial size has been investigated as a link to stroke and it has been found that an association between left atrial diameter and left atrial volume indexed to body surface area and ischemic stroke exists [12-15]. This association is irrespective of the presence or absence of hypertension as a risk factor. This data however does not categorize the independent risk associated with stroke subtypes, as pathological determinants of cardioembolic stroke depend on cardiac morphology more strongly than atherothrombotic stroke do. Our data shows a statistically significant association of mean LAVi and LAD with cardioembolic stroke. This is an important resolution as these echocardiographic determinants may predict the risk of cardioembolic stroke and specific interventions could be offered to this subset of patients, where the diagnosis isn’t apparent. Further investigation for atrial fibrillation (associated with atrial dilation) via Holter and a TEE could be considered in lieu of clinically important decisions pertaining to anticoagulation. Use of left atrial appendage velocity measurement on TTE and or TEE may be a useful surrogate marker of altered appendage function. It could potentially be incorporated in assessing risk of thrombus formation. It may thus, assist secondary prevention decisions, in absence of documented LAA clot or atrial fibrillation. However, these tests are technically demanding in stroke patients with impaired swallowing mechanisms and cognitive impairment.

We must clarify that LAA velocity on TTE is measured by using non-conventional views and has been validated by only a few studies using TEE which is the gold standard. TTE is further limited in good image acquisition in obese, very thin or COPD patients [16-19].

Of particular interest, was the trend in left atrial enlargement and its predilection for the stroke subtype. It was observed that at mild increases in left atrial volume indices, the risk was higher for atherothrombotic strokes but as the left atrial volume index rose higher and entered the moderate to severe category the risk shifted towards the cardioembolic subtype. We hypothesize that these atherosclerotic stroke patients have left atrial enlargement mediated through hypertension. This may also explain the observation that some patients present with cardioembolic strokes but often have underlying atherosclerotic intracranial disease thus displaying a mixed pathogenic mechanism of stroke. However this observation is speculative and requires further investigation via longitudinal investigations in these patients that may involve serial echocardiograms and prolonged arrhythmia monitoring.

With respect to ventricular parameters, poor left ventricular ejection fraction and global left ventricular hypokinesia are direct measures of a poor cardiac reserve and cardiac failure. These conditions lead to vascular stasis and the propensity to thrombus formation in the left ventricle. As a result we saw a higher prevalence of presence of left ventricular thrombus in our patients with varying ventricular dysfunctions thus showing a predilection for development of cardioembolic stroke. Increased left ventricular mass indexed to body surface area is a measure of the left ventricular hypertrophy as a consequence of underlying hypertension. Our data suggests that patients with cardioembolic ischemic strokes have significantly higher LVMi than the atherosclerotic stroke patients. Interestingly the left atrial diameter index and left ventricular mass index in both sexes, although within normal ranges, were also significantly higher in the cardioembolic stroke group than the atherosclerotic stroke group.

The strengths of this study include its robust and complete methods of evaluation and case ascertainment, homogeneity of review, and availability of data on all patients. CCS classification been used as the stroke category classification with experienced investigators and was detailed and blinded to advanced/ cardiologic parameters. There are few investigations that link these advanced parameters to stroke subtypes and we demonstrate that we can use these data to assist in understanding stroke mechanisms in our patients, or further our investigations to ensure that atrial mediated events are not the proximate cause of stroke in our patients. Our study has limitations, this is a retrospective design, a prospective longitudinal assessment would be ideal. In those patients that had atherosclerosis plus and embolic looking stroke with high atrial size, we did not routinely check for atrial fibrillation via prolonged monitoring beyond the hospital stay, this would further cement our preliminary observations, classification was done by two neurologists with one arbitration in case of discordant coding to resolve issues, but a single observation would have minimized the inter observer bias. Although attempts at standardized classification have been made, it must be appreciated that stroke classification isn’t absolute and patients may be have more than one stroke subtype responsible for their event. Furthermore, atrial size increases with higher prevalence of atrial fibrillation this may explain the tilt that we have observed towards cardioembolism. We must add that these are preliminary observations and will require further analysis of association. Lastly, since this is a South Asian population, it has a higher number of patients with atherosclerotic mechanisms mainly intracranial atherosclerosis which although a similar demographic to China, Japan, South East Asia, Hispanics and Blacks does not exactly mirror the stroke subtype distribution of the Western population and these ethnic variations must be kept in mind as these data may not represent global associations.

We suggest that further prospective observational studies studying the relationship between atrial parameters and stroke subtypes should be performed as they provide useful insight into unraveling mechanisms of strokes in individual patients especially those with perhaps multiple overlapping proximate causes of stroke, with increasing atrial dilation. This insight will eventually contribute to better and effective secondary prevention in individual patients.

## Conclusion

Left atrial size may be associated with specific stroke phenotype, further studies investigating atrial parameters on echo cardiogram and stroke subtypes are needed.

## Abbreviations

LAVi: Left atrial volume index; LAD: Left atrial dilation; LVMi: Left ventricular mass index; LA diameter: Left atrial diameter; ECG: Electrocardiogram; TTE: Transthoracic echo; PWT: Posterior left ventricular wall thickness; IVST: Interventricular septal thickness; LVM: Left ventricular mass; ASE: American society of echocardiography.

## Competing interests

The authors declare that they have no competing interests.

## Authors’ contributions

QS: Drafted the first skeleton of the manuscript and ran the final analysis on SPSS, BA: Assisted with all analysis and statistical review, contributed to final writing MA: Assisted with data collection, literature review, SPSS entry and demographic charts, JM, MA, AA: Performed chart review and demographic data collection of the patients through medical record review, FM: Senior Cerebrovascular Research Fellow, CCS certified, performed stroke sub typing, FSA: Provided cardiologic oversight and input and reviewed this manuscript, MK: Senior Cerebrovascular Research Fellow, CCS certified, performed stroke sub typing, AKK: Conceived the study, designed the questionnaire, directed the data entry and review, analyzed the results, reviewed and wrote, formatted and corrected the manuscript in its entirety. All authors read and approved the final manuscript.

## Pre-publication history

The pre-publication history for this paper can be accessed here:

http://www.biomedcentral.com/1471-2377/13/149/prepub
